# Is Zika virus outbreak a solved issue in Brazil?

**DOI:** 10.1590/S1679-45082018ED4325

**Published:** 2018-04-06

**Affiliations:** 1 Faculdade de Medicina, Universidade de São Paulo, São Paulo, SP, Brazil; 2 Hospital Sírio-Libanês, São Paulo, Brazil

Zika vírus (ZIKV) was isolated for the first time in 1947 from a sample of a non-human primate identified at Zika forest in Uganda.^(^ ^1^ ^)^ This is a ribonucleic acid (RNA) virus in the Flaviviridae family that also includes Dengue and Yellow Fever.^(^ ^2^ ^,^ ^3^ ^)^

Although the virus is primarily transmitted through the bite of mosquitoes from Aedes genus,^(^ ^4^ ^)^ it has also been isolated from human body fluids, such as semen and saliva.^(^ ^5^ ^-^ ^7^ ^)^ Sexual transmission of the virus has been increasingly reported, including men to women, men to men, and women to men transmission cases.^(^ ^8^ ^-^ ^15^ ^)^ In addition, possible disease transmission through blood transfusion has also been reported.^(^ ^16^ ^-^ ^18^ ^)^

Human cases of Zika virus infection have been described since 1956,^(^ ^19^ ^)^ but the disease has gained epidemiologic importance after an outbreak and epidemic dissemination of the virus in the Micronesia Islands^(^ ^4^ ^,^ ^20^ ^-^ ^22^ ^)^ and in South and Central America countries.^(^ ^23^ ^,^ ^24^ ^)^

Although the first cases of Zika virus infection in Brazil were confirmed only in the first semester of 2015, recent publications suggest that the disease is probably among us since the beginning of 2014.^(^ ^25^ ^)^ Brazil played an important scientific role and has been internationally recognized within the last two years for reporting the emergency situation of Zika virus outbreak, and also for the identification of neurologic outcomes in infants exposed to the infection during gestation.^(^ ^23^ ^)^ The disease received striking visibility from the scientific community, emphasized by the increase in related publications within the last years.

However, in the last months Zika virus infection seems to be no longer considered a relevant threat among healthcare providers and, more importantly, to the non-expert public. After the great media coverage of the disease in 2015 and 2016, in the year of 2017 little information was broadcasted about Zika – exception made to the substantial attention given to the fact that the incidence of Zika and others arboviruses such as Dengue and Chikungunya declined abruptly in Brazil.^(^ ^26^ ^,^ ^27^ ^)^

In 2015 the Brazilian Ministry of Health advised women to avoid becoming pregnant,^(^ ^28^ ^)^ and in 2016 Zika virus was declared a Public Health Emergency of international concern by the World Health Organization^(^ ^29^ ^)^ resulting in a reduction of birth rates in some regions.^(^ ^30^ ^)^ In 2017, however, the number of consultations in fertilization clinics increased again (Glina and Alvarenga, personal communication), which may reflect a decrease in levels of concern of general population regarding Zika virus infection.

Of note, this expressive reduction in the levels of concern about Zika virus infection in our setting can be premature, if not mistaken. Statistical modeling studies have been used to predict regions around the world that might be more affected by Zika virus. Such predicting models are important not only to guide preventive measures and help plan allocation of therapeutic resources, but are also important to guide where projects should concentrate efforts to clarify a number of unknown aspects of the disease. Modeling studies use information such as presence, efficiency and density of disease vectors, population density, temperature and local humidity, altitude, immunity or susceptibility of resident population, history of incidence of others arboviruses and population movements among geographic regions.^(^ ^31^ ^,^ ^32^ ^)^ Many regions in Brazil remain signposted as high risk areas for Zika occurrence,^(^ ^31^ ^)^ including the state of São Paulo, where available surveillance data show that disease incidence was lower compared with the Northeast region of the country,^(^ ^26^ ^)^ and a large proportion of the population remains susceptible to the infection.

The arboviral transmission season in Brazil is just around the corner, and the risk for Zika virus infection must not be neglected, specially among women at reproductive age and pregnant women.
